# Supplementary material for: Encapsulation boosts islet-cell signature in differentiating human induced pluripotent stem cells via integrin signalling
Source: Sci Rep. 2020 Jan 15;10:414. doi: 10.1038/s41598-019-57305-x (PMC6962451; doi:10.1038/s41598-019-57305-x)
Supplement: Supplementary file 1 — Supplemental Figures and legends. [file 41598_2019_57305_MOESM1_ESM.pdf]

Encapsulation boosts islet-cell signature in differentiating human induced pluripotent stem cells via integrin signalling

Thomas Aga Legøy[1,#], Heidrun Vethe[1,#], Shadab Abadpour[2,3], Berit L. Strand[4], Hanne Scholz[2,3], Joao A. Paulo[5], Helge Ræder[1,6] Luiza Ghila[1] & Simona Chera[1,*]

[1]Department of Clinical Science, University of Bergen, Bergen, Norway

[2]Hybrid Technology Hub-Centre of Excellence, Faculty of Medicine, University of Oslo, Norway

[3]Institute for Surgical Research and Department of Transplant Medicine, Oslo University Hospital, Oslo, Norway

[4]NOBIPOL, Department of Biotechnology and Food Science, Norwegian University of Science and Technology, Trondheim, Norway

[5]Department of Cell Biology, Harvard Medical School, Boston, MA, USA

[6]Department of Pediatrics, Haukeland University Hospital, Bergen, Norway

# These authors contributed equally to this work

* Correspondence to:

Simona.Chera@uib.no

Tel: +47 55 97 12 62
Haukelansbakken 15
5021 Bergen, Norway

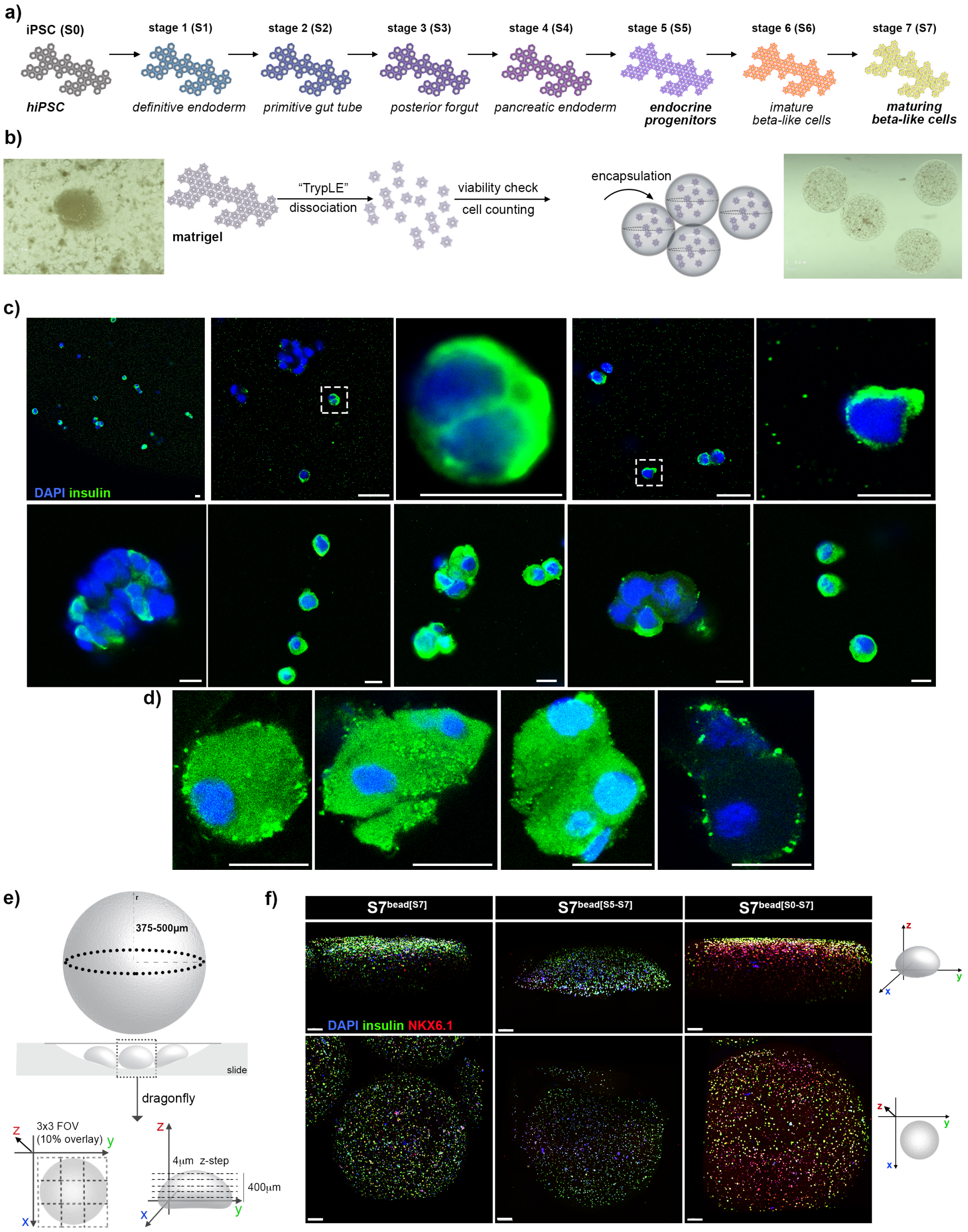

Supplemental Figure 1. a) Scheme depicting the seven stages of the hiPSC differentiation protocol. b) Experimental design of the encapsulation procedure. c) High magnification confocal images of encapsulated cells inside alginate capsules following whole mount immunofluorescence (insulin – green, DAPI – blue). d) High magnification confocal images of encapsulated cells following alginate capsule cryosectioning and immunofluorescence staining for insulin (green) and DAPI (blue). e) Scheme depicting the procedure for the imaging of the encapsulated cells with the Dragonfly confocal, f) 3D reconstructions of insulin (green), NKX6.1 (red), DAPI (blue) immunofluorescence on whole alginate beads containing the three distinct populations analyzed. Scale bar: c, d - 10µm, f - 150µm.

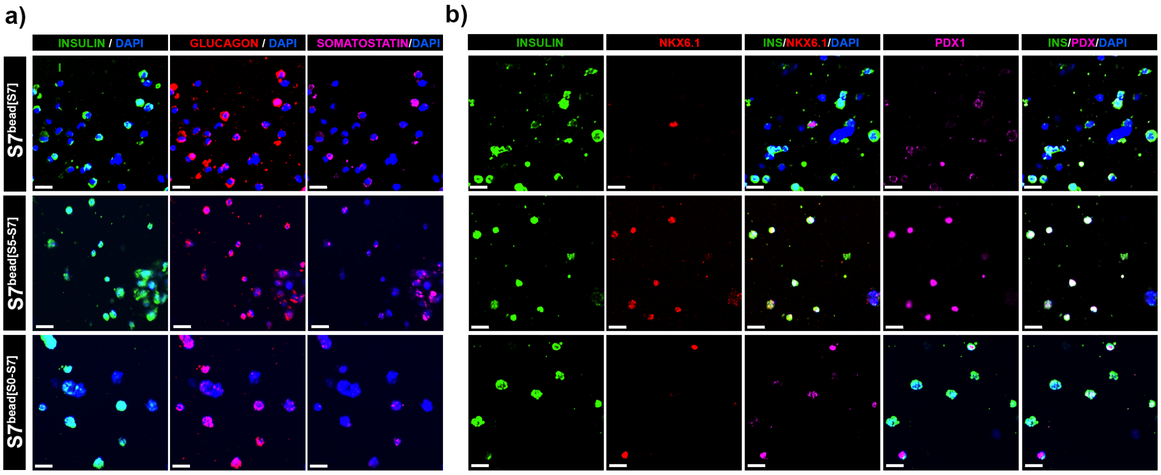

Supplemental Figure 2. a) Immunofluorescence staining of insulin (green), glucagon (red) and somatostatin (purple), DAPI (blue) of the three distinct populations analyzed. b) Immunofluorescence staining of insulin (green), NKX6.1 (red) and PDX1 (purple), DAPI (blue) of the three distinct populations analyzed. Scale bars: 20μm, gamma correction 0.4.

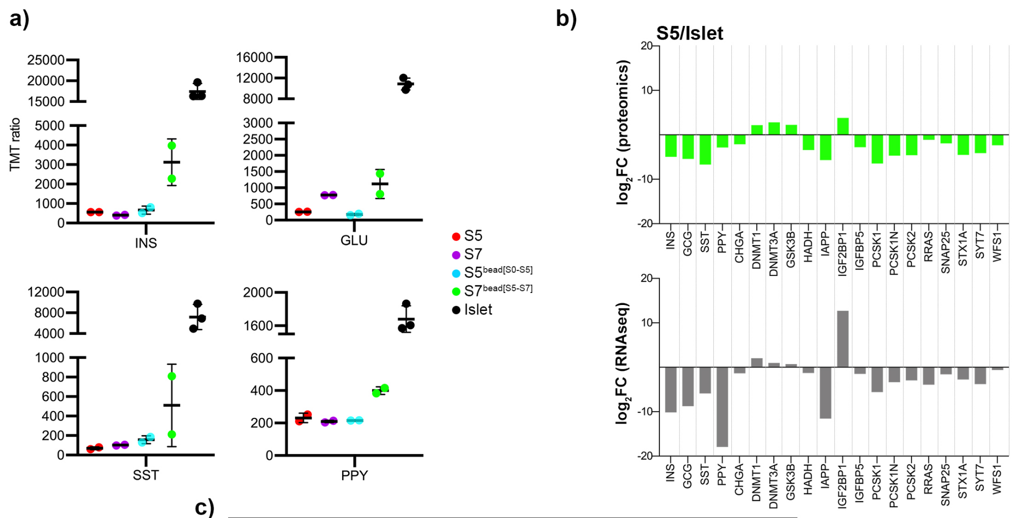

c)

Comparison	Discriminated by
S5-cells/I vs S7-cells/I	Stage/Differentiation cocktail
S5-cells/I vs S5bead[S0-S5]	Encapsulation
S5-cells/I vs S7bead[S5-S7]	Stage/Differentiaton cocktail & Encapsulation
S7-cells/I vs S7bead[S5-S7]	Encapsulation

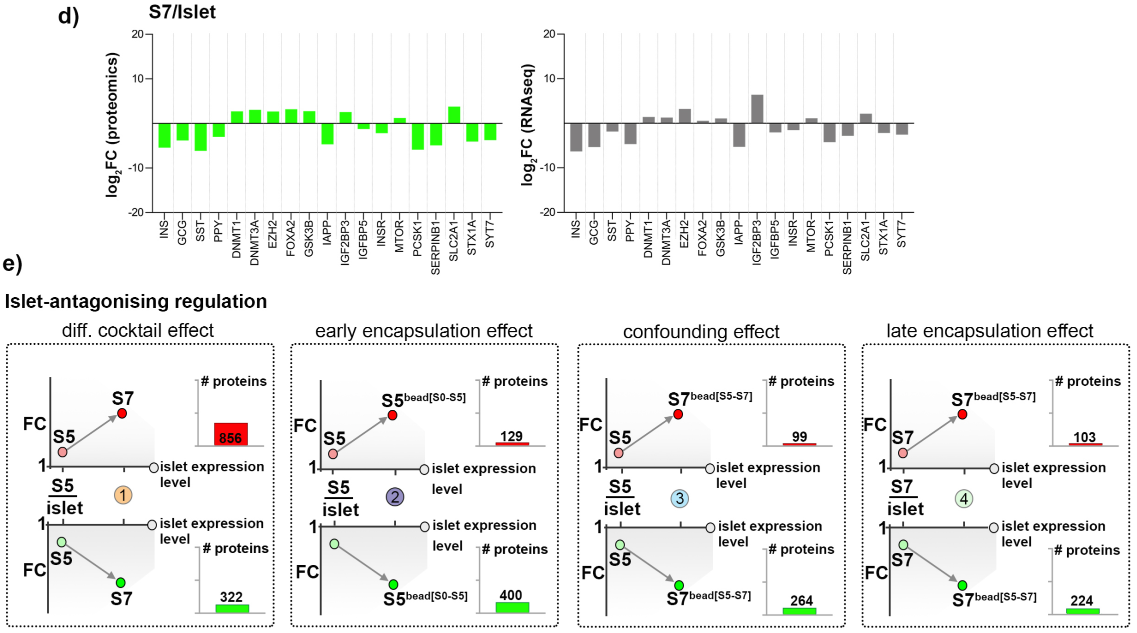

Supplemental Figure 3. **a)** The TMT-ratios of the four main pancreatic hormones in the five conditions analyzed. Graphs data are shown as mean ± SEM. b) Comparison of the proteomics and RNAseq regulation dynamic of β–cell markers in S5-cells as compared to the islet standard (log_2FC). c) Table listing the biological process responsible for the difference between the samples compared. d) Comparison of the proteomics and RNAseq regulation dynamic of β–cell markers in S7-cells as compared to the islet standard (log_2FC). e) The number of proteins showing a dynamic of regulations compatible with an islet-antagonizing pattern in response to each of the four effects considered. Arrows depict the generic prerequisite direction of regulation for group inclusion.

a) **DIFFERENTIATION COCKTAIL EFFECT**

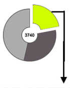

TOP PREDICTED UPSTREAM
TRANSCRIPTION REGULATORS

NAME	activation z-score	prediction
MYC	-3.904	Inhibited
SRF	-3.197	Inhibited
REL	-3.023	Inhibited
SREBF1	-2.923	Inhibited
SREBF2	-2.734	Inhibited
RB1	3.368	Activated
PML	2.186	Activated
RBL1	2.000	Activated
NEUROG1	2.000	Activated

b) **DIFFERENTIATION COCKTAIL EFFECT**

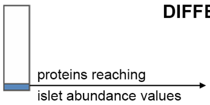

TOP CANONICAL PATHWAYS	
Tumoricidal Function of Hepatic NK Cells	1.99E-04
Mitochondrial Dysfunction	4.74E-04
IGF-1 Signaling	5.14E-04
Tight Junction Signaling	1.18E-03
Valine Degradation I	1.32E-03
Isoleucine Degradation	1.01E-04

TOP PREDICTED UPSTREAM REGs

NAME	p-value
HNF4A	4.84E-07
TP53	3.26E-06
KRAS	9.54E-06
MMP3	6.41E-05
E2F1	2.17E-04

c) **CONFOUNDING EFFECT**

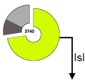

TOP PREDICTED UPSTREAM
TRANSCRIPTION REGULATORS

NAME	activation z-score	prediction
DNAJB6	-2.412	Inhibited
FOXO4	-2.138	Inhibited
HOXC8	-2.000	Inhibited
RB1	3.704	Activated
CDKN2A	2.928	Activated
NFE2L2	2.885	Activated
HES3	2.646	Activated
KLF15	2.641	Activated
SOX4	2.621	Activated
NEUROD1	2.590	Activated
TFEB	2.575	Activated
MAF	2.408	Activated

d) **CONFOUNDING EFFECT**

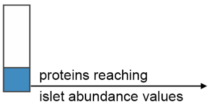

TOP CANONICAL PATHWAYS	
Protein Ubiquitination Pathway	4.48E-15
EIF2 Signaling	4.74E-09
NER Pathway	2.59E-07
Estrogen Receptor Signaling	1.05E-06
Hereditary Breast Cancer Signaling	2.32E-06
PTEN signaling	4.23E-06
mTOR signaling	4.83E-06
PI3K/AKT Signaling	8.26E-06
Molecular Mechanism of Cancer	8.68E-06
Reg. of EIF4 and p70S6K Signaling	9.06E-06
Insulin Receptor Signaling	1.69E-05

TOP PREDICTED UPSTREAM REGs

NAME	p-value
HNF4A	7.61E-18
CST5	3.11E-10
MYC	1.49E-09
RICTOR	3.42E-09
MYCN	6.68E-09

e) **EARLY ENCAPSULATION EFFECT**

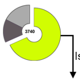

TOP 5 PREDICTED UPSTREAM
TRANSCRIPTION REGULATORS

NAME	activation z-score	prediction
TCF7L2	-4.814	Inhibited
E2F1	-4.529	Inhibited
MYC	-4.224	Inhibited
CTNNB1	-3.928	Inhibited
FOXM1	-3.926	Inhibited
NUPR1	3.796	Activated
CDKN2A	2.635	Activated
PML	2.621	Activated
PDX1	2.512	Activated
MYCN	2.369	Activated

f) **EARLY ENCAPSULATION EFFECT**

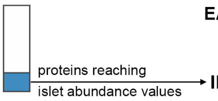

TOP CANONICAL PATHWAYS	
Regulation of eIF and p70S6K Signaling	8.05E-09
EIF2 Signaling	3.29E-07
FAK Signaling	3.33E-07
Cell Cycle Reg. by BTG Family Proteins	5.71E-07
tRNA Charging	9.75E-07
mTOR Signaling	2.45E-06
Rac Signaling	2.77E-06

TOP PREDICTED UPSTREAM REGs

NAME	p-value
HNF4A	1.91E-15
RICTOR	5.39E-09
MMP3	7.22E-08
MYC	3.22E-06
CST5	5.73E-06

g) **LATE ENCAPSULATION EFFECT**

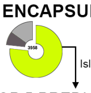

TOP 5 PREDICTED UPSTREAM
TRANSCRIPTION REGULATORS

NAME	activation z-score	prediction
E2F1	-4.671	Inhibited
MYC	-4.067	Inhibited
FOXM1	-3.807	Inhibited
MITF	-3.803	Inhibited
TBX2	-3.273	Inhibited
NUPR1	4.279	Activated
CDKN2A	3.899	Activated
RBL1	2.896	Activated
RB1	2.669	Activated
NCOA1	2.632	Activated

h) **LATE ENCAPSULATION EFFECT**

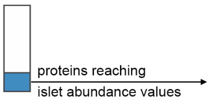

TOP CANONICAL PATHWAYS	
Protein Ubiquitination Pathway	1.14E-15
EIF2 Signaling	3.50E-13
NER Signaling	3.48E-09
Regulation of eIF and p70S6K Signaling	5.01E-06
mTOR Signaling	9.50E-06

TOP PREDICTED UPSTREAM REGs

NAME	p-value
HNF4A	7.61E-18
CST5	3.11E-10
MYC	1.49E-09
RICTOR	3.42E-09
MYCN	6.68E-09

Supplemental Figure 4. Tables depicting the top predicted upstream transcription regulators in response to Differentiation Cocktail Effect. b) Pathways analysis of the proteins reaching abundance levels indistinguishable from the ones detected in native human islets in response to Differentiation Cocktail Effect, c) Table depicting the top predicted upstream transcription regulators in response to Confounding Effect, d) Pathways analysis of the proteins reaching abundance levels indistinguishable from the ones detected in native human islets in response to Confounding Effect, e) Table depicting the top predicted upstream transcription regulators in response to Early Encapsulation Effect, f) Pathways analysis of the proteins reaching abundance levels indistinguishable from the ones detected in native human islets in response to Early Encapsulation Effect, g) Table depicting the top predicted upstream transcription regulators in response to Late Encapsulation Effect, h) Pathways analysis of the proteins reaching abundance levels indistinguishable from the ones detected in native human islets in response to Late Encapsulation Effect.

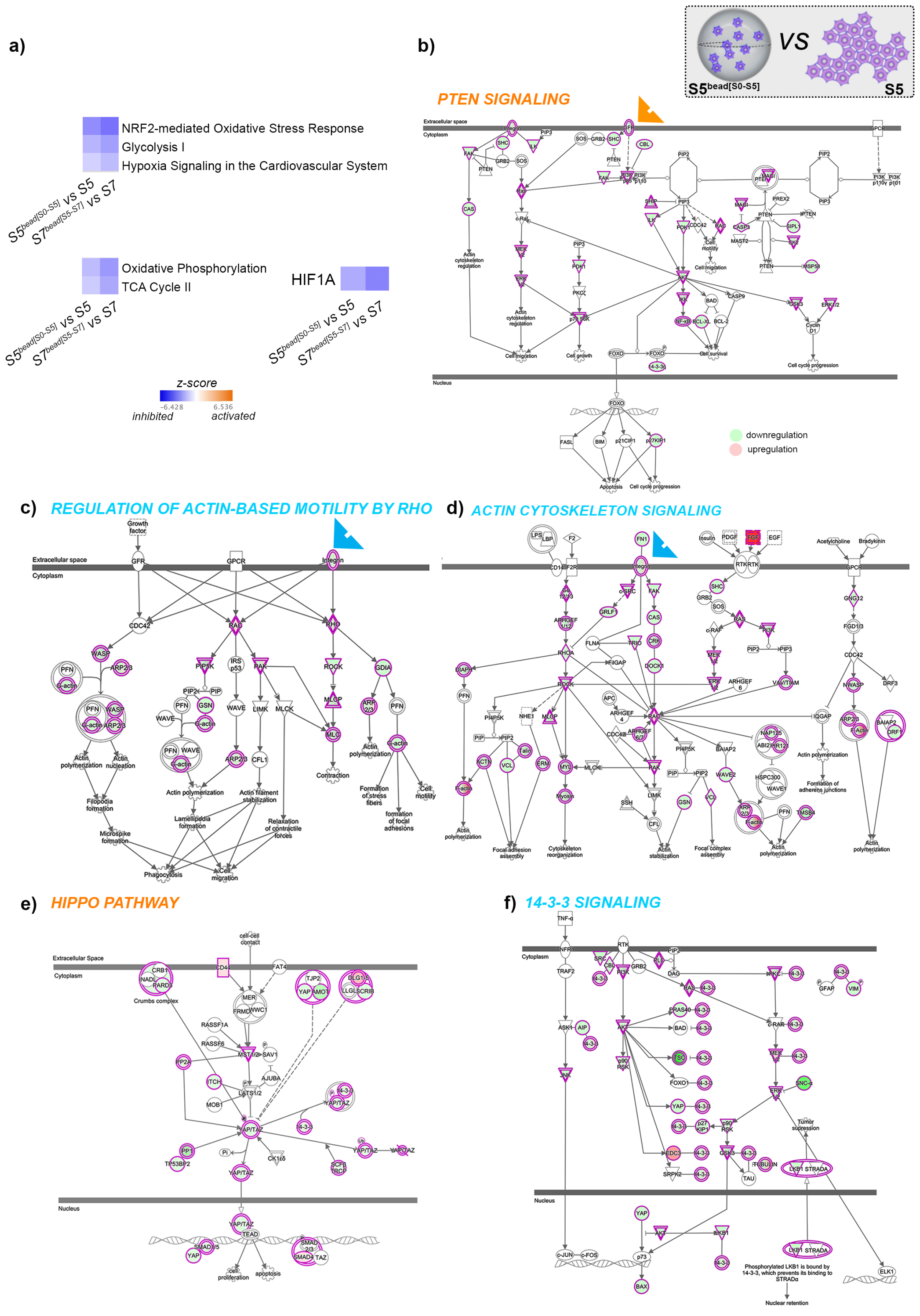

Supplemental Figure 5. a) Comparative pathway analysis of several relevant hypoxia-related pathways and regulators between the overall early- ($S5^{bead[S0\text{-}S5]}$ vs S5) and late-encapsulation ($S7^{bead[S5\text{-}S7]}$ vs S7) effects. b-f) Graphical representations of selected top activated canonical pathways and their observed protein occupancy characterizing the direct comparison between S5-cells differentiated on Matrigel (S5) and S5-cells differentiated in alginate capsules ($S5^{bead[S0\text{-}S5]}$): b) PTEN Signaling, c) Regulation of Actin-Based Motility By Rho Signaling, d) Actin Cytoskeleton Signaling, e) Hippo Signaling, f) 14-3-3 Signaling. (blue - predicted inhibited, orange – predicted activated, green – observed downregulation, red – observed upregulation, arrow heads point to integrin involvement). The comparative analysis and networks were generated through the use of IPA (QIAGEN Inc., https://www.qiagenbio-informatics.com/products/ingenuity-pathway-analysis)

.

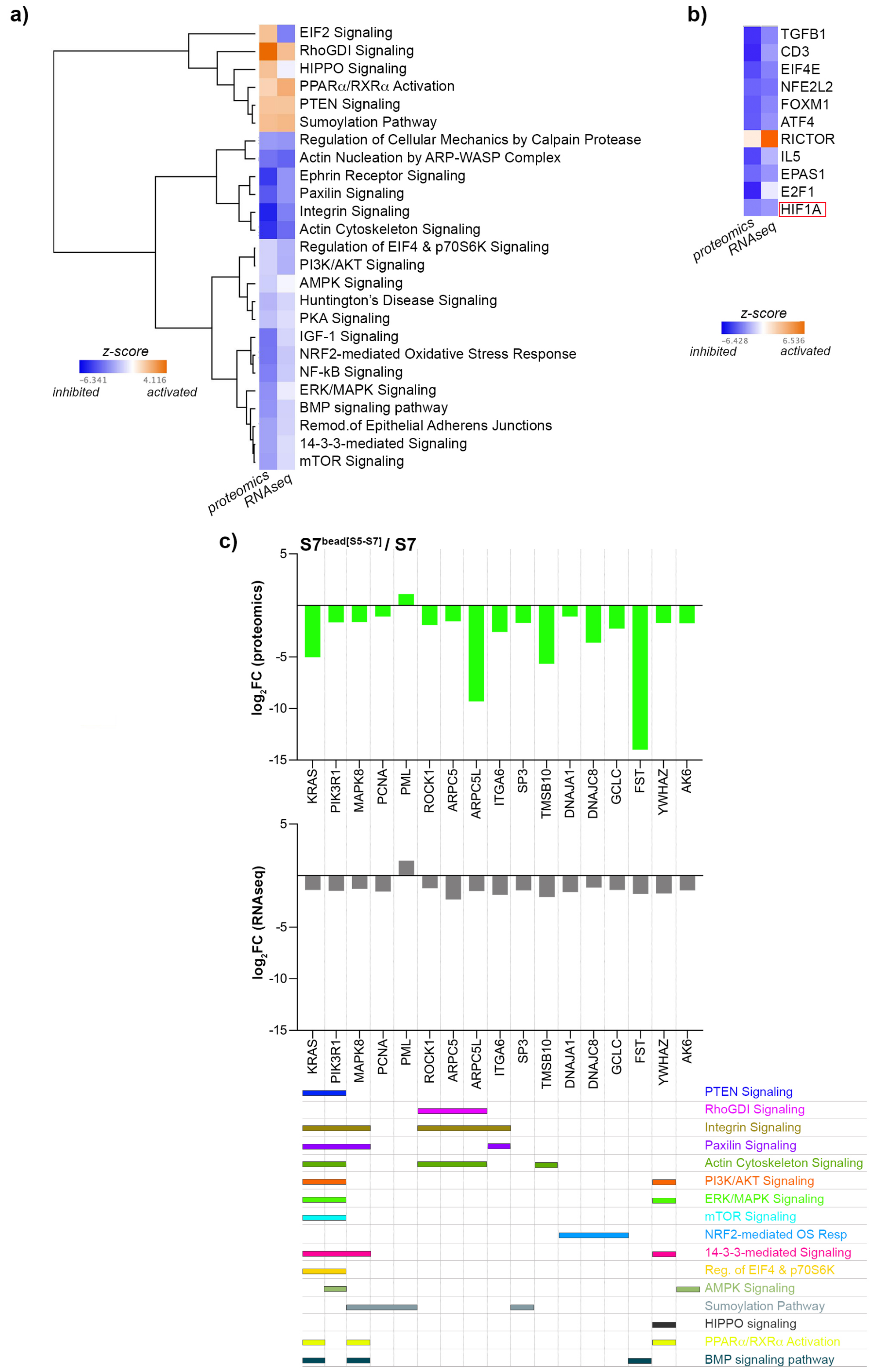

Supplemental Figure 5. a) Comparative pathway analysis of the proteome and RNAseq landscapes characterizing the overall late encapsulation effects. ($S7^{bead[S5-S7]}$ vs S7): a) top canonical pathways (sort method: hierarchical clustering) b) predicted upstream regulators (sort method: trend and score) c) Comparison of the proteomics and RNAseq regulation dynamic of selected key molecular members of the top signaling pathways (log_2FC). The color code bellow shows the involvement of the marker in the corresponding pathway. The comparative analysis was generated through the use of IPA (QIAGEN Inc., https://www.qiagenbio-informatics.com/products/ingenuity-pathway-analysis)
